# Primary adrenal mantle cell lymphoma mimicking a non‐functional retroperitoneal paraganglioma

**DOI:** 10.1002/ccr3.8887

**Published:** 2024-06-02

**Authors:** Kia Seyed Toutounchi, Amirreza Jabbaripour Sarmadian, Amir Vahedi, Seyed Ziaeddin Rasihashemi

**Affiliations:** ^1^ Department of Cardiothoracic Surgery Tabriz University of Medical Sciences Tabriz Iran; ^2^ Connective Tissue Diseases Research Center Tabriz University of Medical Sciences Tabriz Iran; ^3^ Department of Pathology Tabriz University of Medical Sciences Tabriz Iran

**Keywords:** adrenal gland, case report, lymphoma, mantle cell lymphoma, paraganglioma

## Abstract

**Key Clinical Message:**

Patients presenting with abdominal pain and retroperitoneal mass in radiographic images may be in the early stages of primary adrenal mantle cell lymphoma, which requires histological studies for a definite diagnosis.

**Abstract:**

This report presents a 37‐year‐old woman complaining of ambiguous abdominal pain, with imaging findings revealing a retroperitoneal abdominal mass on the left side of the aorta, and a possible diagnosis of non‐functional retroperitoneal paraganglioma. Total laparoscopic excision was performed. Surprisingly, histological examinations revealed features in favor of mantle cell lymphoma.

## 1 INTRODUCTION

Mantle cell lymphoma (MCL) is a rare, aggressive, and heterogeneous subtype of non–Hodgkin's B‐cell lymphoma, accounting for approximately 6% of all lymphomas.^1^, ^2^, ^3^ Accurate and prompt diagnosis is crucial due to its rapid progression nature and poor long‐term prognosis.^1^, ^3^ What delays the diagnosis is the varied clinical manifestations; some patients are asymptomatic, some have minimal symptoms with nodal or extranodal involvements, and some present with general lymphadenopathy, cytopenia, splenomegaly, and systemic involvement.^2^ Therefore, a definitive diagnosis usually requires a comprehensive medical history, physical examination, laboratory workup, tissue biopsy, cytogenic assessment, and immunophenotyping by flow cytometry.^1^, ^2^ This report presents a case of MCL in a 37‐year‐old woman who came to the surgery outpatient clinic complaining of ambiguous abdominal pain, with imaging findings revealing a retroperitoneal abdominal mass mimicking a non‐functional retroperitoneal paraganglioma.

## 2 CASE HISTORY/EXAMINATION

A 37‐year‐old woman came to the surgery outpatient clinic complaining of ambiguous abdominal pain on the left flank. The pain started intermittently about 3 years ago, and during this period, it gradually increased. There was no mention of any other complaints, such as fatigue, fever, weight loss, changes in bowel habits, and changes in menstrual habits. On the past medical history, only asthma was reported for 12 years, which was under control. No family history of diseases, recent surgery or trauma, and no smoking history were reported.

Vital signs were within normal range, and the patient was in a normal state of consciousness. Blood pressure was 125/80 mm Hg; heart rate was 88 bpm; body temperature was 36.7°C; respiratory rate was 18; and oxygen saturation was 95% without oxygen supplementation. On physical examinations, there was a mild tenderness to the deep touch on the left upper and lower quadrants, and there was no evidence of splenomegaly or lymphadenopathy.

## 3 METHODS (DIFFERENTIAL DIAGNOSIS, INVESTIGATIONS, AND TREATMENT)

Laboratory blood tests, including complete blood cell count, serum biochemistry profile, and lactate dehydrogenase (LDH), were requested, all of which were within the normal range, and did not lead us to a specific diagnosis (WBC = 5200/mm^3^, Hb = 13.2 g/dL, PLT = 272,000/mm^3^, INR = 1 Unit, PTT = 31 s, Na = 140 mEq/dL, *K* = 4.6 mEq/dL, urea = 24 mg/dL, creatinine = 0.76 mg/dL, LDH = 438 Unit/L, and ESR = 7 mm/h).

**FIGURE 1:**
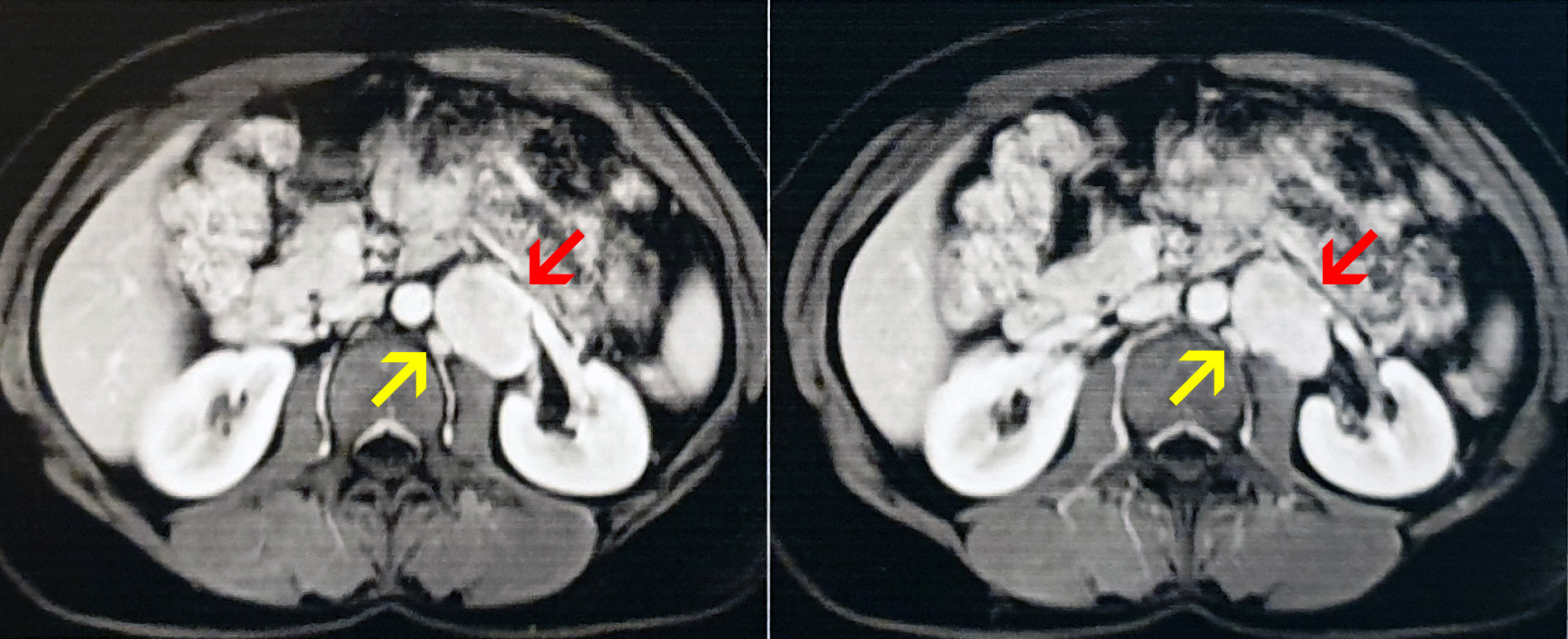
Abdominal CT scan with intravenous contrast shows iso‐dense mass (red arrow) and para‐aortic lymph node (yellow arrow).

Abdominal ultrasonography was requested, which showed a hypoechoic mass with specified margins of dimensions 41 × 28 × 35 mm without calcification in the left para‐aortic region medially to the left kidney. Computed tomography (CT) scan with intravenous contrast was requested to make better decisions about mass, which showed an iso‐dense soft tissue mass of 41 × 26 × 36 mm and para‐aortic lymphadenopathy, as shown in Figure 1. Due to the location of the mass, adrenal masses and paraganglioma (PG) were considered for differential diagnoses. Therefore, adrenal laboratory tests were requested, all of which were within the normal range (AM cortisol = 15 μg/dL, ACTH = 30.90 pg/mL, aldosterone = 6 pg/mL, renin activity = 8.30 mIU/mL, urine epinephrine = 2.1 μg/24 h, urine norepinephrine = 28.8 μg/24 h, urine metanephrine = 86 μg/24 h, urine normetanephrine = 83 μg/24 h, urine vanillylmandelic acid = 5.4 μg/24 h, urine random 5‐hydroxyindoleacetic acid = 5 μg/24 h). As a result, the possibility of non‐functioning masses was raised.

**FIGURE 2:**
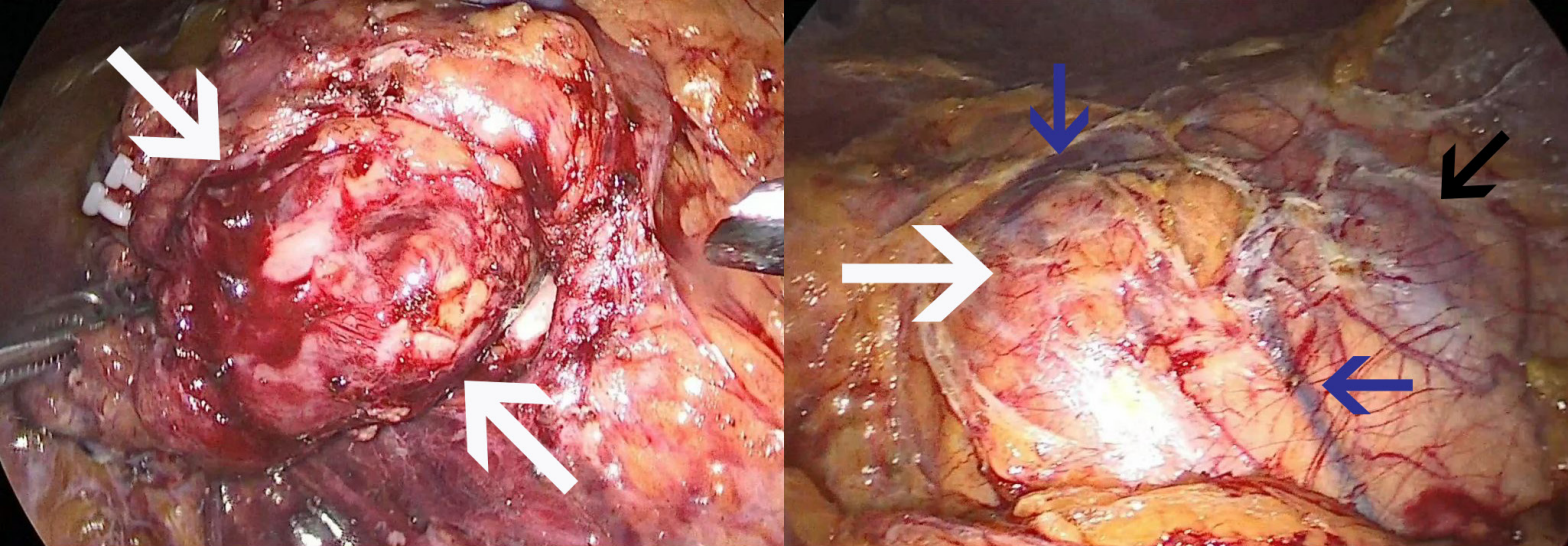
Photographs during Laparoscopy—The anatomical location of the mass (white arrows) is in the vicinity of the left kidney (black arrow), surrounded by renal vessels (blue arrows).

**FIGURE 3:**
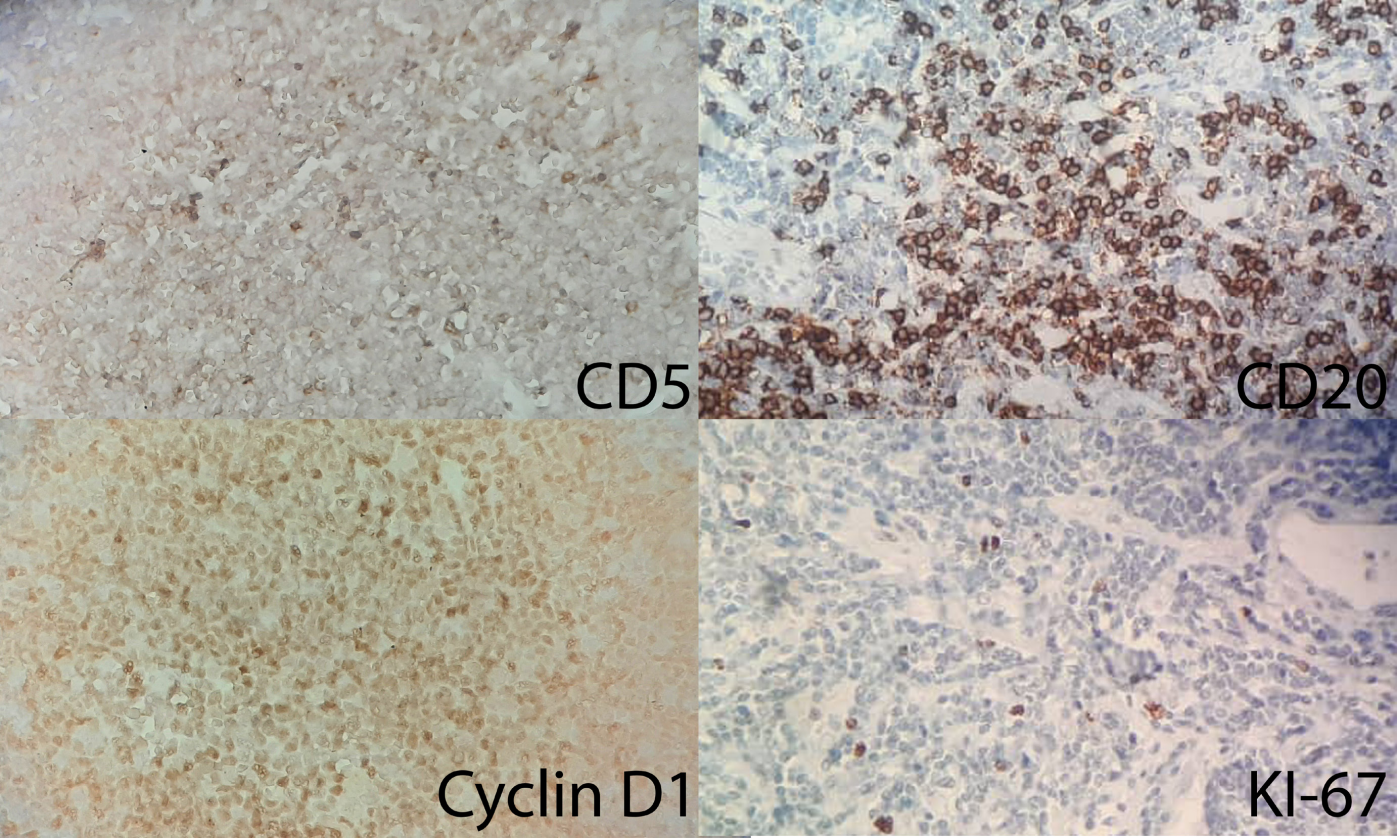
Histological examinations—CD5, positive membranous staining; CD20, positive membranous staining; cyclin D1, diffuse nuclear staining; and KI‐67, positive membranous staining.

A biopsy was planned, but it was not performed due to its challenging anatomical position, and was unsuccessful. So, total excision was performed due to the risk of malignancy after complete preoperative examinations. The patient underwent total laparoscopic excision of the mass, as shown in Figure 2. Histological examinations revealed features in favor of MCL, including partial involvement of mantle zone hyperplasia, stained with CD5, CD20, Cyclin D1, and Ki‐67 as shown in Figure 3.

## 4 OUTCOME AND FOLLOW‐UP

According to the histological results and the final diagnosis of MCL, the patient was discharged in good and stable general condition with a referral to the oncology clinic.

## 5 DISCUSSION

In this report, the patient presented with ambiguous abdominal pain, and in the imaging studies, a suspicious mass with a lymph node was observed on the left side of the abdominal aorta in the area of the left suprarenal. The primary and frequently considered differential diagnosis is adrenal incidentaloma, referring to masses or tumors (≥1 cm in diameter) discovered in the one or both adrenal glands during medical imaging that is not done to evaluate adrenal disease,^4^, ^5^ which in our case was conducted to evaluate abdominal pain without adrenal related symptoms. All patients should be evaluated for pheochromocytoma and excess cortisol secretion through clinical signs and symptoms and biochemical tests.^4^ Our patient did not mention any history of headache, tachycardia, excessive sweating, or palpitation, and all biochemical tests were within the normal range. Moreover, primary hyperaldosteronism should be considered in patients with hypertension and/or hypokalemia,^4^, ^5^ which was not present in our patient and aldosterone was within the normal range. In suspicious cases, biopsy or adrenalectomy should be performed to accurately determine the nature of the mass, such as adenoma, pheochromocytoma, metastasis, and adrenocortical carcinoma.^6^

PG is a rare neuroendocrine neoplasm originating from ganglia along the sympathetic and parasympathetic chain.^7^, ^8^, ^9^, ^10^ It is a chromaffin cell tumor, histologically similar to pheochromocytoma, both with an annual incidence of 0.6 per 100,000, but with the difference that pheochromocytoma is an adrenal tumor and PG is the extra‐adrenal one located at various sites.^7^, ^11^ PG is characterized by catecholamine overproduction, similar to pheochromocytoma, with the most common symptoms being episodic headaches, sweating, and palpitations.^7^, ^9^ However, approximately 10%–15% of PG tumors are non‐functional and asymptomatic or present with nonspecific symptoms, with normal levels of catecholamines in the urine and blood, often found incidentally in imaging as a homogenous round or oval solid mass along the sympathetic chain, mainly in the abdomen associated with the abdominal aorta.^8^, ^9^, ^10^ The location of the mass, which was along the left sympathetic chain associated with the abdominal aorta, and the normality of the laboratory findings suggested a non‐functional retroperitoneal paraganglioma.

As mentioned, for a definitive diagnosis, histological studies are needed. In this case, biopsy was not performed due to its challenging anatomical position, so total excision was performed due to the risk of malignancy. It should be noted that laparoscopic resection of PG is considered challenging due to its critical anatomic location and proximity to the abdominal aorta, so laparotomy is preferred.^8^ However, laparoscopic resection was successfully performed in this patient. Surprisingly, histological examinations revealed features in favor of MCL.

MCL is usually seen in patients over the age of 60 and is more common in men.^1^, ^2^ However, our case was a 37‐year‐old woman. Histologically, the characteristic immunophenotype of MCL cases includes co‐expression of CD5, B‐cell antigens (CD19, CD20, CD22, and CD24), and cyclin D1 overexpression,^2^, ^12^ which in our case were positive for CD5, CD20, and cyclin D1. Moreover, Ki‐67 index is generally associated with a more aggressive course,^2^ which was positive in our patient. In addition, MCL is associated with chromosomal translocation t(11;14) (q13;q32),^1^, ^2^, ^3^ which we did not study in our case. Most MCL patients are diagnosed with advanced disease stages and systemic involvement, leading to a poor prognosis, and only 10% of patients present with localized nodal or extranodal involvement,^1^, ^13^ similar to our case. In cases in the early stages of the disease, partial involvement of the mantle zone has been reported,^13^ similar to our case.

Primary adrenal lymphoma (PAL) is a rare extranodal involvement, which is very rare with MCL.^14^ In about 70% of cases, it is bilateral,^15^ unlike our case, which was unilateral and it is often misdiagnosed as adrenal tumors. PAL is more common in male patients over 60 years old, which is in contrast to our case. Patients usually present with abdominal pain, similar to our case, and increased levels of LDH,^16^ which was normal in our case. It may also lead to adrenal insufficiency, which was normal in our case. PAL generally has a good prognosis if detected as soon as possible by a combination of medical history, laboratory workup, imaging findings, and histological studies.^14^, ^15^, ^16^

In conclusion, patients presenting with abdominal pain and retroperitoneal mass in radiographic images may be in the early stages of primary adrenal mantle cell lymphoma, which requires laboratory studies to evaluate and exclude adrenal disorders, and histological studies for a definite diagnosis.

## AUTHOR CONTRIBUTIONS

**Kia Seyed Toutounchi:** Data curation; investigation; writing – original draft. **Amirreza Jabbaripour Sarmadian:** Writing – original draft; writing – review and editing. **Amir Vahedi:** Conceptualization; data curation; validation. **Seyed Ziaeddin Rasihashemi:** Conceptualization; methodology; project administration; supervision.

## FUNDING INFORMATION

There was no financial support or funding for this research.

## CONFLICT OF INTEREST STATEMENT

The authors declare no financial and non‐financial competing interests related to this work.

## ETHICS STATEMENT

This study was performed according to the principles outlined by the World Medical Association's Declaration of Helsinki on experimentation involving human subjects, as revised in 2000, and was approved by the Tabriz University of Medical Sciences ethics committee with the approval number IR.TBZMED.REC.1402.322 on 2023/07/17.

## CONSENT

Written informed consent was obtained from the patient to publish this report in accordance with the journal's patient consent policy.

## Data Availability

Data are available from the corresponding author on reasonable request.
